# Nurses’ and patients’ experiences and preferences of the ankle-brachial pressure index and multi-site photoplethysmography for the diagnosis of peripheral arterial disease: A qualitative study

**DOI:** 10.1371/journal.pone.0224546

**Published:** 2019-11-07

**Authors:** Jason Scott, Jan Lecouturier, Nikki Rousseau, Gerard Stansby, Andrew Sims, Lesley Wilson, John Allen

**Affiliations:** 1 Faculty of Health and Life Sciences, Northumbria University, Newcastle upon Tyne, United Kingdom; 2 Institute of Health and Society, Newcastle University, Newcastle upon Tyne, United Kingdom; 3 Northern Vascular Centre, Newcastle upon Tyne Hospitals NHS Foundation Trust, Newcastle upon Tyne, United Kingdom; 4 School of Surgical and Reproductive Sciences, Newcastle University, Newcastle upon Tyne, United Kingdom; 5 Northern Medical Physics and Clinical Engineering Department, Newcastle upon Tyne Hospitals NHS Foundation Trust, Newcastle upon Tyne, United Kingdom; 6 Institute of Cellular Medicine, Newcastle University, Newcastle upon Tyne, United Kingdom; Plymouth State University, UNITED STATES

## Abstract

Peripheral arterial disease is a global health problem, affecting around 20% of people aged over 60 years. Whilst ankle-brachial pressure index (ABPI) is regularly used for diagnosis, it has a number of limitations, which have presented a need for alternative methods of diagnosis. Multi-site photoplethysmography (MPPG) is one such method, but evidence of acceptability of both methods is lacking. This study aims to describe and compare preferences and experiences amongst nurses and patients of ABPI and MPPG use in primary care. We used qualitative research methods in the context of a clinical diagnostic study comparing ABPI with MPPG. Use of ABPI and MPPG by 13 nurses were observed with 51 patients across general practice surgeries in North-East England in 2015/16. Follow-up semi-structured interviews were conducted with 12 nurses and 27 patients. Data were thematically analysed. Two major themes were identified: (1) *device preferences*; (2) *test discomfort and anxiety*. There was a compelling preference for MPPG due to ease of use, speed of the test, patient comfort, and perceived device accuracy/objectivity. However some patients struggled to identify a preference, describing ambivalence to medical testing. ABPI was deemed uncomfortable and painful, particularly when the blood pressure cuff was inflated at the lower limbs. There was also evidence of anxiety amongst patients when their foot pulses were not identified using ABPI. Whilst ABPI is a non-invasive and routine procedure it was associated with a number of drawbacks in clinical practice. Nurses required considerable dexterity to employ the test, and it resulted in anxiety amongst some patients. Conversely, MPPG was deemed to be easier and quicker to use, and perceived to be less subjective. Should diagnostic accuracy and cost be comparable to ABPI, then the findings of this study suggest MPPG would be preferable to ABPI for patients as well as nurses.

## Introduction

Peripheral arterial disease (PAD) is the restriction of blood flow in the arteries, typically in the lower extremities, the most common symptom of which is intermittent claudication (pain) [1, 2], though PAD can also contribute to functional impairment without intermittent claudication such as atypical exertional leg pain [2]. Other symptoms include ischaemic rest pain, ischaemic ulceration, and limb loss in the most extreme cases [3]. PAD is a global health problem, affecting around one in five people aged over 60 years [4]. People with PAD have been identified to have a significantly lower quality of life and high levels of pain [5], although PAD is often under-reported, under-diagnosed and sub-optimally treated [6, 7]. More specifically, the majority of people with PAD are asymptomatic [4] despite similar levels of mortality to symptomatic patients [8], and many of those patients who are symptomatic do not present to their general practitioner or other healthcare professional due to a lack of knowledge about PAD [9]. This is despite PAD being associated with a high risk of other vascular events such as heart attacks and strokes, as well as limb amputation and claudication [10, 11].

PAD can be diagnosed using various different methods; guidelines for the National Health Service (NHS) in England [12] include verbal communication of symptoms that indicate intermittent claudication and critical limb ischaemia, physical examination of legs and feet for evidence of critical limb ischaemia, examination of femoral, popliteal and foot pulses, measuring the ankle brachial pressure index (ABPI), or a duplex ultrasound arterial scan. Other methods include digital subtraction angiography [13], magnetic resonance angiography, and computed tomography angiography [14].

Within primary care settings, ABPI is used often because it is able to be conducted by trained nursing staff, and it can demonstrate relatively high levels of accuracy [15]. ABPI works by measuring blood pressure non-invasively in the arteries which supply the lower extremities, typically at the level of the ankle, and comparing with blood pressures measured at the arm [16]. Recent evidence suggests that ABPI is a cost-effective method of screening for PAD as an indicator of cardiovascular risk [17] despite it being relatively time consuming due to a required ten minute rest period prior to testing. However, whilst ABPI is able to detect severe disease, it is less accurate at detecting mild or moderate disease [18]. ABPI is also liable to large variation in practice; the position of patient, order of limb measurement and use of mean or maximum measurements in the ABPI calculation can all differ depending on the clinician conducting the test [19], possibly due to there being no standard approach to training [20]. Another explanation for variation in ABPI measurement is terminal digit preference, where clinicians have been identified to overuse specific digits, particularly the digit 0, when recording systolic and diastolic blood pressure test results [21].

These limitations of ABPI suggest a need for alternative approaches to identifying and diagnosing PAD, with more novel ways of doing so currently being developed. One novel approach to diagnosing PAD utilizes multi-site photoplethysmography (MPPG) technology, which is a non-invasive and simple-to-use test for PAD that measures how long it takes the patient’s pulse to reach different parts of their body. This is based on optical peripheral pulse waveform analysis, and demonstrates similar sensitivity and specificity in detecting PAD as ABPI [22–24]. However, due to its novelty, there is currently no published data on its usability or acceptance within primary care. ABPI and MPPG were chosen for comparison as ABPI is the standard approach to measuring PAD in primary care practice, and both approaches follow a similar process (see Box 1).

**Box 1. Description of clinical diagnostic accuracy study, *Novel pulse device for diagnosis of PAD* (NOTEPAD), and comparison of measurement processes for ABPI and MPPG technology used in the study**

To assess diagnostic accuracy of MPPG, patients with symptomatic PAD and an equal number of age-matched non-PAD patients were identified and recruited from general practice registers in the North East of England. A total of 306 patients were recruited into the trial and all study measurements were carried out in primary care. Informed consent was taken by a vascular research nurse who carried out a screen for PAD symptoms using the Edinburgh Claudication Questionnaire, recorded the participant's height and weight to calculate BMI and recorded basic demographics and past medical history and medications. Study MPPG and ABPI measurements were then carried out by a practice nurse. All practice nurses had been previously trained in the two techniques by the study team. ABPI used as a comparator as it is deemed standard practice in primary care, and it was measured using standard methods as recommended by NICE guidelines [12]. Finally, a vascular scientist blinded to the ABPI and MPPG results carried a bilateral lower limb Duplex vascular ultrasound scan to act as a gold standard test for the presence of PAD.

ABPI measurement process, adapted from National Institute for Health and Clinical Excellence [12] guidelines on diagnosing and managing PAD:

- Patient should be lying down, following a rest period of ten minutes
- Systolic blood pressure readings should be recorded for both arms and feet (posterior tibial, dorsalis pedis) using an appropriately sized cuff
- Measurements taken manually using a Doppler ultrasound probe of suitable frequency in preference to an automated system
- Calculate the index in each leg by dividing the highest ankle pressure by the highest arm pressure.

MPPG measurement process (adapted from Allen *et al. [23]*) for *Novel pulse device for diagnosis of PAD* (NOTEPAD) diagnostic trial:

- Patient should be lying down, following a rest period of ten minutes
- Pulse probes clipped bilaterally to the ear lobes, index finger pads and great toe pads
- Gains adjusted for a clear pulse obtained at each measurement site; the patient is asked to remain comfortably still throughout their measurement
- Pulses recorded for about a minute and the individual site MPPG Shape Index measures of PAD automatically and immediately calculated and displayed for the operator to note down.

The aim of the study was to describe and compare nurses’ and patients’ experiences and preferences of ABPI and MPPG use in primary care. The study was situated in a larger clinical diagnostic study comparing ABPI with MPPG (see Box 1 for description of the wider study).

## Methods

Qualitative design methods (semi-structured interviews and observations) were incorporated into the NOTEPAD study described in the box. Such an approach has the ability to develop a better understanding of factors that contribute to increased or decreased effectiveness of interventions in the real world [25]. Furthermore, it has been reported that embedded qualitative research in trials is able to provide insight into how trial participants experience the intervention [26]. In this study, the insight was extended beyond only patients receiving the intervention to also include nurses using ABPI and MPPG. The data is based on transcripts produced from audio recordings of interviews with research participants. We do not have consent to disseminate the full transcripts and we wish to respect the anonymity of the research participants. Restrictions on data sharing were in place following ethical review by NHS Research Ethics Committee North East–Newcastle & North Tyneside 1 (ref: 14/NE/1238). However, relevant sections of the transcripts have been included in the manuscript to support our findings. Study materials are available as supporting materials (S1–S5 Files) to enable replication, and we would be willing to interrogate the dataset on behalf of other studies upon reasonable request. Requests should be made to the study sponsor by emailing Angela Topping at nuth.nuthsponsorship@nhs.net.

### Setting and sampling

A convenience sample of nursing staff, including practice nurses and research nurses, from 16 primary care practices who were involved in the wider clinical diagnostic study (NOTEPAD) were invited to participate in semi-structured interviews. For observations, nursing staff and patients from nine practices were sampled based on their involvement in the NOTEPAD study. These nine practices were sampled as the team felt they provided diversity in terms of geographical spread. No practices declined to be observed. A convenience sample of patients who had been observed were also invited to participate in interviews based upon their participation in the NOTEPAD study and having been observed. This sample included patients previously diagnosed with PAD (case patients) who were recruited to the NOTEPAD study through local PAD registers (the Quality and Outcomes Framework [27] states that general practices should establish and maintain a register of patients with PAD (pages 35–36)), and age-matched patients who had no diagnosis of PAD (control patients). Data collection (observations and interviews) was conducted up until data saturation (the point at which new data collection was not adding to or elaborating upon the themes identified in the analysis) was reached [28]. The number of interviews within each subgroup of participant (nurses, case patients and control patients) either meeting or exceeding the required number identified as being necessary for saturation [29].

### Recruitment and consent

Nurses conducting both ABPI and MPPG, which included practice nurses and research nurses, were invited to participate in semi-structured interviews and observations of the two tests in use. Nurse consent to be observed was obtained during recruitment to the diagnostic accuracy study. Nurse consent to participate in a semi-structured interview was obtained separately, either before or after observation.

Patients were recruited to take part in the observational component of the study by a research nurse prior to the patients’ appointments for the diagnostic accuracy study tests. Upon completion of both tests, patients were then given information about the semi-structured interview, and contacted a minimum of 48 hours later by a researcher to take consent. Interviews were only conducted with patients who had been observed undergoing the ABPI and MPPG tests. We did not collect details of people who declined to participate.

### Data collection

Non-participant observations were conducted by two researchers (JS, male and KL, female) to explore nurses’ behaviours and responses when using ABPI and MPPG, and how patients responded to the tests. Researcher observations occurred within treatment rooms of the primary care practices and of training sessions for nurses on the use of ABPI and MPPG. Clinics consisting of morning or afternoon timeslots, with up to four patients per clinic, were held specifically for the purpose of the clinical diagnostic accuracy study. The majority of the clinics consisted of one nurse conducting the tests, but on some occasions there were two nurses. Prior to the start of the clinic, the researcher would present to the nurse(s) and agree where to be positioned within the treatment room so to cause as little interference as possible but maintaining a position to observe both the nurse(s) and patient. Hand-written observation notes were taken by the researcher and typed up into full notes following the observation.

Semi-structured interviews were conducted by JS and KL, with nurses and case and control patients. Topic guides (S6 and S7 Files) were developed to reflect the objectives of the study by the study team, which included input by LW who has over 20 years of experience as a cardiovascular nurse, and also the study patient and public involvement group. Nurse topic guides focused on the nurse’s professional background, the training they received for the diagnostic accuracy study, their perspectives on ABPI and MPPG, and thoughts on the future use of MPPG in primary care. Topic guides for control patients included the participant’s experience of being in the diagnostic accuracy study, their perspectives on ABPI and MPPG, and their preference for either test. Topic guides for case patients were the same, but also included questions on their PAD, including their history of PAD, the diagnosis of PAD, and how their PAD was being managed. Interviews were conducted at a time and place convenient to the participant, with only the researcher and participant present. For nurses, this was always during working hours at their primary care practice or by telephone. For patients, this was always during the day at their home or by telephone. All interviews were audio-recorded and transcribed verbatim by a third party transcription company. A researcher checked the transcriptions for accuracy.

### Data analysis

Data were analysed using thematic analysis following the stages described by Braun and Clarke [30]. This included familiarisation with the data set by one researcher (JS), which was obtained from both collecting the data and reading transcripts. Initial inductive codes were then generated, using NVivo 10 to catalogue the codes. Data analysis sessions were held, at which source data and the developing coding frame were discussed and refined by JS, JL, and NR. JS then generated the final themes, which were then discussed and refined further by JS, JL, and NR. Data from all sources was triangulated at the point of coding, with all data coded into the same themes.

### Validity and rigour

Several established approaches were taken to ensure the validity and rigour of the findings [31], including development of a coding system, peer review of themes, triangulation of multiple data sources (nurse interviews, patient interviews and observations), and provision of thick description that recognises the context of data collection, supported by quotes and detailed field notes. Furthermore, the *Consolidated criteria for reporting qualitative research* (COREQ; S8 File) checklist [32] was used to confirm complete and transparent reporting of qualitative data, ensuring sufficient information is provided to determine transferability of findings.

### Ethical considerations

Ethical and governance approvals were granted by an NHS Research Ethics Committee North East–Newcastle & North Tyneside 1 (ref: 14/NE/1238) and the MHRA (ref: CI/2015/0017). The trial was registered 13^th^ October 2016 (ISRCTN ref: 13301188).

## Results

Data were collected between June 2015 and September 2016 and included 51 observations of ABPI and MPPG in use at nine GP practices by 13 nurses. Twenty-nine observations were of case participants, and 22 were of control participants. Data also included semi-structured interviews with 12 nurses, 17 case patients and ten control patients. Nurse interviews lasted between 16m 24s and 34m 22s (average 26m 09s). Patient interviews lasted between 10m 01s and 36m 46s (average 18m 44s).

The findings are presented below according to the two major themes and their sub-themes identified in the analysis; (1) *device preferences*, and (2) *test discomfort and anxiety*. Selective representative quotes have been included.

### Device preferences

The first major theme identified related to participants’ device preferences, in particular a compelling preference among nurses for MPPG over ABPI. This is despite the MPPG device that was tested not being feature-complete, including requiring manual adjustment of gains to take readings. Preference for MPPG amongst both nurses and patients tended to be determined by four aspects; ease of use for nurses, speed of test, comfort, and perceived device accuracy and objectivity. These are succinctly summarised by one nurse:

*“I think the fact that [MPPG is] very quick*, *it can be done very quickly for the patients*, *it’s not as painful with the fact that you’re not blowing up the cuff on the patient*, *and sometimes when you’re using the Doppler [ultrasound] machine trying to find the pulse rate is really hard*. *I just think it’s very effective*. *If it’s proven that the data [from the study] is matching with the ABPI that we do*, *it just proves it’s a very effective piece of equipment*. *It’s easy to use*. *It’s easier for the patients and its pain free*. *It’s just a more pleasant experience for the patient*.*”* (Nurse-3 interview)

#### Ease of use for nurses

All of the nurse participants that used ABPI and MPPG stated a preference for MPPG, often citing the ease of use. This largely related to the required dexterity of inflating the blood pressure cuff whilst simultaneously holding the Doppler ultrasound probe in place, where sometimes the smallest of movements would result in losing the required signal location. Nurses quickly became familiar with the device, despite it being a prototype design, and found it much easier to use than ABPI.

*“I initially thought the [prototype MPPG] machine was quite fiddly*, *just with all the leads*. *It looked*, *initially*, *quite frightening because of all the leads and stuff*. *But once you got to use it*, *it was just confidence I think*, *and practice with it*. *I found the MPPG machine more accurate and easier to use than the ambulatory blood pressure*.*”* (Nurse-10 interview)

*“During the break I talk to the nurse about how it was (doing the tests) with the first patient*, *the first time she’d done it*. *She says that the MPPG was much easier than the ABPI but she was expecting it the other way around*. *She thought there were more cables for MPPG than there actually are*.*”* (Observation-12 field notes, 2^nd^ participant)

#### Device accuracy and objectivity

Another reason given by nurses for preferring MPPG over ABPI was its perceived accuracy. Whilst at the time of data collection it was not known whether MPPG would be as accurate as ABPI, nurses still had more confidence in the device’s reliability, acknowledging that ABPI was to some extent subjective; *“I think the Doppler is much more open to interpretation by the clinician*.*”* (Nurse-2 interview). This perception was not unique to nurses conducting the tests. In the following quote, a patient reflects on their experience of ABPI both within and prior to the diagnostic accuracy study setting.

*“I mean*, *if your new test [MPPG] proves to be accurate*, *I’d much prefer that than the Doppler*. *[…] I mean*, *if you get a nurse that’s not fully qualified on the Doppler*, *she can miss the pulse*, *you know*. *Either by A*, *going in the wrong place or B*, *not being quite able to find it*. *If they’re going to use the Doppler [ABPI]*, *perhaps there should be two tests like the previous one we just had [in the study]*. *You know*, *do one and then get perhaps somebody else to do another one or give you a break and then do it again to ensure that you’re actually getting an accurate reading*.*”* (Patient-16 interview, case)

#### Comfort

As already identified, discomfort or pain was present for some patients whilst receiving the ABPI test, in particular when the blood pressure cuff was expanded on lower limbs. Knowing that MPPG was discomfort and pain free was also a contributing factor for nurses’ preference for MPPG, one of whom described it as *“less invasive for the patient”* (Nurse-10 interview), and also for patients’ preference for MPPG. Notably the following patient quote also presents uncertainty about their preference, which was relatively common amongst patients and is discussed in depth later in the results section.

*“Aye I don’t know*. *I mean having the*, *having the (MPPG) clips was*, *was no discomfort where I suppose you have a bit of discomfort with the cuffs but I don’t think there’s*, *you know*, *I don’t think I’d have a preference actually*, *as long as it was done that would be it*. *Yeah*, *I*, *I think the new one is possibly better*, *you know*, *the clips is possibly better ‘cause you don’t have the discomfort that you have with the*, *the cuffs*.*”* (Patient-21 interview, case)

#### Speed of test

Both nurses and patients also recognised that MPPG was quicker, regardless of whether the rest period time was included or not. Notably, this preference was also clear even where (dis)comfort was not a contributing factor to a patient’s preference.

*“neither were uncomfortable and*, *but the one with the clips [MPPG] was quicker*. *So I suppose*, *I*, *I suppose the*, *the more modern version*.*”* (Patient 6-interview, case)

*“From the patients' point of view*, *lying for a long time is difficult for some people*. *So if it could be a shorter session where you have to lie flat*, *that would be an improvement for both of them*.*”* (Nurse-11 interview)

The use of the words “more modern version” by Patient-6 was also common amongst nurses when conducting the tests; in particular when introducing the devices, nurses were likely to describe MPPG as *‘the new bit of kit’* (Observation-2 field notes, 3^rd^ participant) or variants of this.

#### Preference uncertainty

As outlined previously, some patients struggled to identify a preference during interviews, even when asked directly. When pressed to choose one or the other, they almost always favoured MPPG but found it difficult to explain why. This appeared to link into the previous finding that tests in general, even if they cause discomfort or pain, are *“part and parcel of the procedure”* (Patient-4 interview, control) and therefore as a patient they will do whatever is required of them, or *“it’s entirely up to what the doctors want to do”* (Patient-12 interview, control).

*“Well the [MPPG] clips was very easy*. *When*, *when the [ABPI cuff] pressure was on*, *one of my ankles*, *there was a little bit of pain*. *But really*, *I*, *I wouldn’t be too bothered whether it was either*. *You know*, *I wouldn’t be too bothered whichever it was*.*”* (Patient-10 interview, case)

There were also occasions when patients were unable to provide a preference at all, even when pressed by the interviewer. One patient was unable to differentiate between the procedure for ABPI and MPPG, suggesting they considered them to be a single event rather than multiple tests, *“And the procedure for the new apparatus just seemed to be exactly the same as the bloody old one”* (Patient-5 interview, case). In later interviews with nurses who had conducted ABPI, they were asked if they knew why some patients may have difficulty identifying a preference. Answers to this question supported the notion that tests were seen as a necessity, as exemplified by the following quote.

*“No*, *I can’t think of why they wouldn’t [have a preference]*. *I think maybe they just accept any test for what it is*, *so those people wouldn’t be bothered what test they did*, *as long as they get the result at the end and they’re able to do something about the result*. *They’re not entirely different really*, *it’s either laying there having a Doppler done or having probes on your ears*, *or ears and fingers and toes*, *you know*, *so they’re probably not that different*.*”* (Nurse-12 interview)

### Test discomfort and anxiety

The second major theme identified related to the relative levels of discomfort and anxiety experienced by both case and control participants whilst having the tests conducted.

It was identified that requiring participants to lie flat, including the rest and test periods, caused discomfort amongst some patients, particularly as they were not routinely offered neck support. As data were collected during a diagnostic accuracy study rather than during natural use of either device, participants were required to lie down for longer than would be normal in order to accommodate both tests. Regardless of this prolonged period of lying flat, it was almost always immediately upon lying down that participants raised concerns. As a result, participants often requested to be sat upright, even if only partially, and study nurses were often hesitant of doing so due to concern that it would influence the results. Despite this, upon the requests, either the beds would be raised slightly, or participants would be provided with a pillow.

*‘One nurse asks the patient to lie down on the bed*. *Patient says she’ll struggle to lie completely flat and asks for her head to be raised–explains that she gets dizzy when completely flat*. *The nurse raises the head part of the bed a small bit*.*’* (Observation-11 field notes, 2^nd^ participant)

This discomfort was also acknowledged by nurses and patients during interviews. One nurse describes how the discomfort can be exacerbated by individual patient characteristics and the types of beds used in primary care:

*“The patients are required to lie completely flat and for some patients that can be very difficult and very uncomfortable*.*[…] the couches aren’t particularly well padded*. *And if the patients are overweight*, *or*, *you know*, *elderly*, *they have neck or spinal problems*, *it can be difficult for the patient”* (Nurse-1 interview)

*“[Lying down] is something a few years ago*, *wouldn’t have been the slightest problem at all*. *But after a few minutes it became distinctly uncomfortable*. *So it*, *it’s difficult to straighten my legs*, *so there was pressure on my knee*, *trying to keep it straight*, *and across my shoulders*. *Because there was no head support*, *was the problem*. *However I just mentioned it to the nurse*, *‘hey up*,*’ I says*, *I said*, *‘this is uncomfortable*,*’ and so she just gave me a cushion for my head*, *and that was end of the problem*.*”* (Patient-5 interview, case)

This discomfort was not unique to either device within the diagnostic accuracy study context, as all patients were required to lie flat at all times. A second type of discomfort was also apparent in relation to ABPI, where the expansion of the blood pressure cuffs around lower limbs caused discomfort and, in several participants, pain. This was also reflected in interviews with nurses and patients, with one nurse reflecting on experiences of conducting ABPI prior to the diagnostic accuracy study:

*“I think [ABPI is] a bit more uncomfortable for the patients as well*, *especially when it goes round their foot*, *round their leg*. *Especially if they’ve got high blood pressure ‘cause you’re pumping it up quite high to*, *to get the beat come in and it’s… I’ve had a few patients in the past who have screamed”* (Nurse-7 interview)

There was no reported or observed difference in pain or discomfort between the case and control participants, and when asked, nurses were rarely aware of any discomfort or pain in specific patients, though some were aware it was a possibility. This was often because they were focusing on obtaining the correct reading, though they acknowledged the potential for pain or discomfort.

*‘When the nurse expands blood pressure cuff [on patient’s lower leg] the patient winces and takes a deep breath*. *The second time for the ankle reading*, *the patient also makes a noise from the pain*. *Nurses don’t appear to notice as they are concentrating on taking the readings’* (Observation-11 field notes, 1^st^ participant)

Despite the pain, patients were quick to dismiss it as a necessary part of healthcare, and rarely informed the nurse that the test was painful or uncomfortable.

*“Well when*, *once it gets tight*, *it’s*, *you feel a bit of discomfort but it only lasts seconds so it’s not too bad*. *It was quite*, *on the calves of the legs*, *it was quite painful*. *But as I say*, *it’s short term so*, *you know*, *it’s short term so it’s nothing to worry about”* (Patient-21 interview, case)

Beyond the physical discomfort, mental anxiety was evident in several patients. In particular, patients showed concern about the test readings when they did not go smoothly, such as a nurse being unable to find the patient’s foot pulse during ABPI. Patients often shrouded this anxiety within a joke. In the following excerpt from field notes, the patient also raises concerns about their general health.

*‘Patient picks up that one of his [ABPI] readings is lower and starts asking about it*. *The research nurse tries to explain that it is normal*, *but he doesn’t seem to believe her; he keeps challenging and asking*, *‘why*?*”* (Observation-12 field notes, 4^th^ participant)

Due to the nature of the MPPG device assessed, where there is no indication of test outcome, most patients did not question the meaning of the test. Where few patients did, nurses explained that they themselves were unaware of the outcome. The same patient that continually questioned the research nurse over his ABPI readings later accepted, without further probing, that the nurse did not know the meaning of his MPPG results, *‘Patient asks what his ‘MPPG’ readings mean and nurse says she doesn’t know*. *Patient comments that every day is a training day’* (Observation-12 field notes, 4^th^ participant).

## Discussion

This is one of the first studies to use qualitative research methods to describe and compare preferences and experiences relating to ABPI and other non-invasive vascular tests for PAD diagnosis in primary care. This is despite qualitative methods being widely used in usability testing for various types of healthcare interventions [33, 34], including imaging technologies [35]. ABPI is generally recognised to be a non-invasive and simple procedure [15, 36], but we identified that ABPI is associated with a number of drawbacks in clinical practice that had not been identified previously, including pain and anxiety for patients, which contributed to an overwhelming preference for the MPPG PAD technology amongst the nurse and patient participants.

Notably, patients demonstrated or reported feelings of discomfort and pain during ABPI, which has not been explored in-depth previously in the literature, nor is it recognised as a limitation of ABPI within guidelines for PAD [12]. Some patients who experienced pain or discomfort in relation to the blood pressure cuff expansion appeared to be ambivalent; not only did they not highlight the pain or discomfort to the nurse conducting the test, but they also later explained that discomfort or pain was an expectation of medical testing, thus perceiving it to be a necessity to receiving a diagnosis [37]. This was emphasised when asked their preference for a device; many patients struggled to identify a preference without further prompting. Whilst there is evidence that invasive tests can impact on patient acceptance and participation, specifically when associated with pain and discomfort [38], there is a need for further research to usefully explore the meanings and experiences of patients undergoing routine non-invasive tests in primary care, especially where mild pain or discomfort is possible. This is of particular relevance for PAD, the symptoms of which are already under-reported [6, 7], and which could be influenced by test anxiety.

The exception to this was where discomfort resulted from lying flat, with patients willing to inform the nurse in order to change their circumstances. One possible explanation for this willingness was that patients were able to anticipate their discomfort of lying down flat based on previous experiences, but, particularly in relation to blood pressure cuffs used on their lower limbs, were unlikely to have experienced the discomfort before. MPPG was perceived to be a quicker test to complete than ABPI, thus reducing the overall time required for patients to be lying down and being exposed to discomfort. Furthermore, nurses also failed to recognise when patients were in discomfort or pain, and so were unable to directly reassure patients or to perform ameliorating actions to reduce the discomfort or pain. Regardless of how patients justified experiencing pain, it is important to recognise that non-invasive tests, such as ABPI, are not pain-free, and patients should be informed of this potential a priori as part of the consent process.

For both ABPI and MPPG, patients sometimes queried the meaning of the test results even when the test was still being conducted. This was particularly evident during ABPI when either the dorsalis pedis or posterior tibial (foot) pulses were difficult or not possible to identify, either due to excessive claudication, the nurse’s ability with the device, or a combination of the two. Patients querying the test results during and immediately after the tests, particularly when a nurse was not qualified to provide a diagnosis, raises practical implications for both tests as it can place the nurse in an awkward position. For instance in practice, whilst the nurse may have a strong indication of the test result, it is not always likely that they have the full clinical picture, and are therefore unable to give the patient an answer. It has been identified that medical waiting periods require patients and clinicians to adopt coping strategies to ease patient anxiety [39, 40]. One such strategy is *preparative waiting*, a part of which requires nurses to provide adjusted and individualised information to patients [41]. MPPG, in its prototype design used in this study, was able to obfuscate any identification of potential symptoms, allowing the test results to be analysed alongside the full clinical picture before being presented to the patient, therefore providing nurses with the opportunity to assist the patient with their coping strategies and reducing the potential for anxiety that has arisen during the test.

A further drawback of ABPI which contributed to nurses’ preference for MPPG over ABPI was that ABPI required considerable dexterity to perform the test effectively. This is an identified barrier within the literature on ABPI, and has been reported to contribute to variations in measurement accuracy [42]. Furthermore, poor technique in performing ABPI in practice has been widely reported [20, 42, 43], and the high level of required dexterity contributed to the subjectivity that study participants associated with ABPI. MPPG was deemed to be a more objective measure as it was not liable to the same limitations, despite the actual device used in this study being a prototype. The ease of use of MPPG also potentially has further practical implications; it is likely to require less training and will not be subject to the same experiential learning curve of ABPI, and it can reduce the time required for diagnosis, all of which may reduce the cost of implementation, though further evidence is required to determine whether it is more cost-effective than ABPI.

Given the current challenges in diagnosing PAD, MPPG was reported by nurses to be easier and quicker to use, and appeared to cause less discomfort or pain in patients. MPPG may therefore help to reduce identified delays to diagnosis [6] and lead to greater uptake of formal diagnostic testing for PAD rather than the tendency to rely upon clinical symptoms [44]. MPPG may also help to reduce anxiety relating to negative test outcomes and could help to improve the patient’s experience of the diagnosis pathway, though additional formal evaluation of the final device will be required, which should include further examination of whether MPPG reduces test anxiety and therefore contributes to improved reporting of PAD symptoms.

### Limitations

No standardised, validated measure of pain or discomfort was used, mainly because based on prior literature and NICE guidelines [12] they were not anticipated. Whilst this would have provided a quantifiable comparison between the two different tests, the evidence provided in this study was conclusive that ABPI resulted in more discomfort than MPPG, which was identified via numerous methods. However, future work (including trials of new medical devices) where discomfort may be expected should consider combining both quantitative and qualitative approaches to identifying and measuring pain or discomfort.

Another limitation was that many of the participating nurses were not regular users of ABPI prior to involvement in this study, although all nurses regardless of prior ABPI experience received full training in the use of both techniques. This may partially explain the drawbacks identified with ABPI and the general preference for MPPG. However, this is not the first study to identify the dexterous nature of ABPI and the negative impact that this has on test outcomes [42], suggesting the results were not artefacts of the study design. Furthermore, the nurses were novices in both devices, and so comparisons made were from an equal perspective.

Finally, data was collected from participants who were taking part in a larger diagnostic study, and so the observations in particular are unlikely to full reflect actual experiences of using the two devices in practice. However, the tests were conducted as closely as possible to natural conditions, with patients seeing their own practice nurse in their own primary care practice. Particularly for MPPG, which used a prototype device design, further work will be required to determine the ease and speed of use of the device should it enter into routine practice, and to explore how it impacts upon the diagnosis pathway for PAD. Sampling for this qualitative study was based on recruitment to the clinical diagnostic accuracy study, and so demographic data was not collected beyond the patients’ PAD diagnosis.

## Conclusion

This is one of the first studies to use qualitative approaches to investigate device preferences and experiences of using medical devices for PAD diagnosis in primary care. Whilst ABPI is a non-invasive and routine procedure, it is associated with a number of drawbacks in clinical practice. ABPI was found to cause discomfort and pain in some patients which is not currently acknowledged in national guidelines, and some patients experienced anxiety when a foot pulse was difficult or not possible to locate. In contrast, MPPG was deemed to be easier and quicker to use, and perceived to be less subjective. Should MPPG be at least comparable to ABPI in diagnostic specificity and sensitivity and with similar device costs, the results of this qualitative study suggest it would be preferable to ABPI for both patients and nurses. Further health technology evaluation of the final MPPG device is expected once implemented into practice on its pathway to adoption.

## Supporting information

S1 File Patient information sheet–interview. (DOCX)

S2 File Participant information sheet–health professionals. (DOCX)

S3 File Participant information sheet–health professionals interviews. (DOCX)

S4 File Participant consent form–health professionals. (DOCX)

S5 File Participant consent form–patients. (DOCX)

S6 File Topic guide–patients–cases. (DOCX)

S7 File Topic guide–interviews health professionals. (DOCX)

S8 File COREQ checklist. (DOCX)
