# Supplementary material for: Nurses’ and patients’ experiences and preferences of the ankle-brachial pressure index and multi-site photoplethysmography for the diagnosis of peripheral arterial disease: A qualitative study
Source: PLoS One. 2019 Nov 7;14(11):e0224546. doi: 10.1371/journal.pone.0224546 (PMC6837749; doi:10.1371/journal.pone.0224546)
Supplement: S1 File — (DOCX) [file pone.0224546.s001.docx]

**Participant Information Sheet (Interviews)**

**Novel pulse device for diagnosis of PAD**

**(NOTEPAD)**

You have kindly agreed to take part in this research study which involves being assessed with a new device called MPPG.

As well as assessing how reliable this new device is, it is very important that we find out what patients think of it. To do this we are asking a small number of patients if they would be happy to be interviewed. This information sheet provides further details*.*

**Why have I been chosen?**

You have been chosen to be interviewed because you have been assessed with the new device, MPPG.

**Do I have to take part in an interview?**

It is up to you to decide whether or not to take part in an interview. Whether you agree to take part or not, this will not affect the standard of care you receive.

**What will happen if I agree to be interviewed?**

We will discuss the study with you and answer any further questions you may have. If you are still happy to go ahead we will arrange a convenient time and place to conduct the interview with you (this could be by telephone if you prefer). Just before starting the interview we will ask you to sign a consent form.

The interview should take no more than an hour, though this will depend upon how much you have to say. It will be conducted by an experienced researcher and audio recorded so they can talk with you without having to make notes. If you want to stop the recording or the interview at any point you are completely entitled to do so.

**Will what I say remain confidential?**

Yes. We are all bound by a written code of confidentiality. Everything you say during the interview will remain strictly confidential. The audio recording will be typed up and the transcript anonymized so that you cannot be recognized. Paper transcripts and audio-recordings will be destroyed when the study ends but transcripts will be stored electronically in a secure password protected computer for 15 years. Only the

researchers and those employed on the study will have access to the records and the transcripts.

**Who is organising and funding the research?**

The study is being organised by the Newcastle upon Tyne Hospitals NHS Foundation Trust and Newcastle University. It is funded by the National Institute for Health Research (NIHR) Invention for innovation (i4i) programme.

**What will happen to the results of the research study?**

The results will be presented at meetings of health professionals and published in medical journals. A report will also be submitted to the funding organisation. A summary of the study will be available to research participants. You will not be identified by name in any reports or publications.

**Who has reviewed this study?**

All research in the NHS is looked at by an independent group of people, called a Research Ethics Committee, to protect your interests. This study has been reviewed and given favorable opinion by Newcastle & North Tyneside 1 Research Ethics Committee.

**What if there is a problem?**

If you have any concerns about the study you can contact the local research team (see below) who will do their best to answer your questions. If you remain unhappy and wish to complain formally, you can do this through the NHS Complaints Procedure. Details can be obtained from the hospital.

**Further Information and Contact Details**

If you have any further questions or need any further information regarding this study, do not hesitate to contact a member of the research team using the contact details below:

Jan Lecouturier Tel: 0191 208 5629

Co-lead, Qualitative Study Email: Jan.lecouturier@ncl.ac.uk

Nikki Rousseau Tel: 0191 208 7162

Co-lead, Qualitative Study Email: Nikki.rousseau@ncl.ac.uk

Jason Scott Tel: 0191 208 8848

Researcher Email: Jason.scott@ncl.ac.uk

Victoria Morgan Tel: 0191 208 6826

Secretary Email: Victoria.morgan@ncl.ac.uk

Sister Lesley Wilson Tel: 0191 2448457

Northern vascular Centre, Freeman Hospital

For independent advice regarding participating in research studies you can contact the Patient Advice and Liaison Service (PALS).

PALS can be contacted on:

**Freephone: 0800 0320202**
**Text: 01670 511098**
**Email: northoftynepals@nhct.nhs.uk**

You can also write to PALS at:

**Freepost: RLTC-SGHH-EGXJ**
**North of Tyne PALS**
**The Old Stables**
**Grey's Yard**
**Morpeth**
**NE61 1QD**
