# Supplementary material for: Nurses’ and patients’ experiences and preferences of the ankle-brachial pressure index and multi-site photoplethysmography for the diagnosis of peripheral arterial disease: A qualitative study
Source: PLoS One. 2019 Nov 7;14(11):e0224546. doi: 10.1371/journal.pone.0224546 (PMC6837749; doi:10.1371/journal.pone.0224546)
Supplement: S2 File — (DOCX) [file pone.0224546.s002.docx]

| **Novel pulse device for diagnosis of PAD** |  |
| --- | --- |

**Participant information sheet**

**(Health Professionals)**

We would like to invite you to take part in a qualitative research study. Before you decide we would like you to understand why this research is being done and what it would involve for you. Talk to others about the study if you wish and please ask us if there is anything that is not clear.

Thank you for taking the time to consider the study*.*

**What is the purpose of this study?**

The purpose of this study is for doctors and nurses in general practice to try out a new device to test circulation in patients who may have peripheral arterial disease. The device, called multi-site photoplethysmography (MPPG), measures how long it takes the patient’s pulse to reach different parts of their body. To conduct the test small blood flow probes are lightly attached to the patient’s big toes, index fingers and ear lobes. We want to find out how and when the device is used and what staff think of it (and of the training that is provided in the use of the device).

In some participating general practices we will also explore patients’ views of being tested with the new device.

**How is this study being done?**

We will collect data through interviews and observations of staff using the device in the general practice setting. Ideally we would like to interview staff just after they have received training and later in the study when they have had a chance to use it.

**Who is doing this study?**

We are a team of researchers based in the Institute of Health and Society at Newcastle University. Our contact details are listed below. This study is funded by the National Institute for Health Research Invention for Innovation research programme.

**How and why have I been picked?**

You have been chosen to take part in this research because your practice has agreed to participate in this study using the new device with patients. You have been identified as a member of staff within the practice who would use the device with patients.

**Do I have to take part?**

No, it is entirely up to you whether you take part in the interviews and observations and no one will put pressure on you to agree to either.

**What happens if I agree to take part?**

If you would like to take part in this study we will ask you to sign a consent form to say that you agree to participate. Agreeing to take part in one aspect of the study does not mean you have to consent to other aspects. You can decide whether to consent to interviews and/or observations.

**If I agree to be interviewed, what will be involved?**

The interview will take place wherever is most convenient to you (e.g. at your place of work, or at the Institute of Health and Society at Newcastle University). A topic guide will be used but staff will be encouraged to talk freely and raise any other issues related to the device and its use. The interview should take no longer than an hour though this will depend upon on how much you have to say. The interview will be conducted by an experienced researcher and recorded so that the researcher can talk with you without having to make notes. If you want to stop the recording or the interview at any point you are completely entitled to do so.

**If I agree to be observed using the device, what will be involved?**

Observation will involve a researcher being present when the device is used with patients. This will only be conducted where patients have also consented to the observation. The researcher will make detailed written notes during the observation. These notes will be typed up and anonymised.

**Will what I say remain confidential?**

Yes. We are all bound by a written code of confidentiality. Everything you say during the interview and when being observed using the device will remain strictly confidential. Transcripts will be anonymised so that you cannot be recognised from any of the information we collect from you. Paper transcripts and audio-recordings will be destroyed when the study ends but transcripts will be stored electronically in a secure password protected computer for 15 years. Only the researchers and those employed on the study will have access to the recordings and the transcripts.

**Has this study been reviewed by NHS Research Ethics Committee?**

Yes Newcastle & North Tyneside 1 Research Ethics Committee has reviewed the study and the study has been given a favourable opinion. This committee is responsible for ensuring that all medical research going on in the area is ethical and fair to study participants.

**Further Information and Contact Details**

If you have any further questions or need any further information regarding this study, do not hesitate to contact a member of the research team using the contact details below:

Jan Lecouturier Tel: 0191 208 5629

Co-lead, Qualitative Study Email: Jan.lecouturier@ncl.ac.uk

Nikki Rousseau Tel: 0191 2087162

Co-lead, Qualitative Study Email: Nikki.rousseau@ncl.ac.uk

Jason Scott Tel: 0191 208 8848

Researcher Email: Jason.scott@ncl.ac.uk

Victoria Morgan Tel: 0191 2086826

Secretary Email: Victoria.morgan@ncl.ac.uk

Sister Lesley Wilson Tel: 0191 2448457

Northern Vascular Centre, Freeman Hospital

For independent advice regarding participating in research studies you can contact the Patient Advice and Liaison Service (PALS).

PALS can be contacted on:

**Freephone: 0800 0320202**
**Text: 01670 511098**
**Email:** [**northoftynepals@nhct.nhs.uk**](mailto:northoftynepals@nhct.nhs.uk)

You can also write to PALS at:

**Freepost: RLTC-SGHH-EGXJ**
**North of Tyne PALS**
**The Old Stables**
**Grey's Yard**
**Morpeth**
**NE61 1QD**

|  |  |  |
| --- | --- | --- |
|  |  |  |
