# Supplementary material for: Nurses’ and patients’ experiences and preferences of the ankle-brachial pressure index and multi-site photoplethysmography for the diagnosis of peripheral arterial disease: A qualitative study
Source: PLoS One. 2019 Nov 7;14(11):e0224546. doi: 10.1371/journal.pone.0224546 (PMC6837749; doi:10.1371/journal.pone.0224546)
Supplement: S3 File — (DOCX) [file pone.0224546.s003.docx]

| **Novel pulse device for diagnosis of PAD** |  |
| --- | --- |

**Participant information sheet**

**(Health Professionals)**

We would like to invite you to take part in a qualitative research study. Before you decide we would like you to understand why this research is being done and what it would involve for you. Talk to others about the study if you wish and please ask us if there is anything that is not clear.

Thank you for taking the time to consider the study*.*

**What is the purpose of this study?**

A new device for diagnosing peripheral arterial disease (PAD) has been developed. The device, called multi-site photoplethysmography (MPPG), measures how long it takes the patient’s pulse to reach different parts of their body. We want to find out how the device could fit into the current pathways for PAD, and what staff think of the device.

**How is this study being done?**

We will collect data through interviews with staff who could use the device in the general practice setting or who have experience of diagnosing and managing PAD.

**Who is doing this study?**

We are a team of researchers based in the Institute of Health and Society at Newcastle University. Our contact details are listed below. This study is funded by the National Institute for Health Research Invention for Innovation research programme.

**How and why have I been picked?**

You have been chosen to take part in this research because your practice has already participated in this study, testing the new device with patients. You have been identified as a member of staff within the practice who could use the device with patients or who has knowledge about PAD pathways.

**Do I have to take part?**

No, it is entirely up to you whether you take part in the interview and no one will put pressure on you to agree.

**What happens if I agree to take part?**

If you would like to take part in this study we will ask you to sign a consent form to say that you agree to participate. Your practice will be reimbursed for your time.

**If I agree to be interviewed, what will be involved?**

The interview will take place wherever is most convenient to you (e.g. at your place of work, or at the Institute of Health and Society at Newcastle University). A topic guide will be used but staff will be encouraged to talk freely and raise any other issues related to the PAD pathway and the device’s potential use. The interview should take no longer than an hour though this will depend upon on how much you have to say. The interview will be conducted by an experienced researcher and recorded so that the researcher can talk with you without having to make notes. If you want to stop the recording or the interview at any point you are completely entitled to do so.

**Will what I say remain confidential?**

Yes. We are all bound by a written code of confidentiality. Everything you say during the interview will remain strictly confidential. Transcripts will be anonymised so that you cannot be recognised from any of the information we collect from you. Paper transcripts and audio-recordings will be destroyed when the study ends but transcripts will be stored electronically in a secure password protected computer for 15 years. Only the researchers and those employed on the study will have access to the recordings and the transcripts.

**Has this study been reviewed by a Research Ethics Committee?**

[Statement of ethics approval to be included once obtained]

**Further Information and Contact Details**

If you have any further questions or need any further information regarding this study, do not hesitate to contact a member of the research team using the contact details below:

Jan Lecouturier Tel: 0191 208 5629

Co-lead, Qualitative Study Email: [Jan.lecouturier@ncl.ac.uk](mailto:Jan.lecouturier@ncl.ac.uk)

Nikki Rousseau Tel: 0191 2087162

Co-lead, Qualitative Study Email: [Nikki.rousseau@ncl.ac.uk](mailto:Nikki.rousseau@ncl.ac.uk)

Jason Scott Tel: 0191 208 8848

Researcher Email: [Jason.scott@ncl.ac.uk](mailto:Jason.scott@ncl.ac.uk)

Victoria Morgan Tel: 0191 2086826

Secretary Email: [Victoria.morgan@ncl.ac.uk](mailto:Victoria.morgan@ncl.ac.uk)

|  |  |  |
| --- | --- | --- |
|  |  |  |
