# Supplementary material for: Nurses’ and patients’ experiences and preferences of the ankle-brachial pressure index and multi-site photoplethysmography for the diagnosis of peripheral arterial disease: A qualitative study
Source: PLoS One. 2019 Nov 7;14(11):e0224546. doi: 10.1371/journal.pone.0224546 (PMC6837749; doi:10.1371/journal.pone.0224546)
Supplement: S4 File — (DOCX) [file pone.0224546.s004.docx]

**Research Participant Consent Form – Health Professionals**

**Novel pulse device for diagnosis of PAD (NOTEPAD)**

| **S** |  |  |  |
| --- | --- | --- | --- |

|  |  |
| --- | --- |

**Practice Number:**

|  | **Please initial** |
| --- | --- |

| 1. I confirm that I have read and understand the information sheet   Version 3.0*,* dated 12 November 2015, for the above study. |  |
| --- | --- |
| 1. I have had the opportunity to read the information, ask questions and have   had these answered to my satisfaction. |  |
| 1. I understand that being observed and taking part in an interview is completely voluntary and a decision not to take part will have no negative consequences. |  |
| 1. Interview - I agree* to the interview being audio recorded. |  |
| 1. Interview - I understand that I am free to stop the interview at any point, and to request the destruction of data for which I am responsible. |  |
| 1. Observation – I agree* to a member of the research team observing me using the PPG device with patients and taking notes. (Consent will also be obtained from the patient). |  |
| 1. Observation – I understand that I am free to stop the observation at any point and to request the destruction of data for which I am responsible. |  |

*** If you do not agree please initial the box and put X next to your initials.**

| 1. I understand that I will not be personally named in any report and that anything I say will be treated in confidence. |  |
| --- | --- |
| 1. I understand that any information collected will be kept in a secure way and   that interview or observational data will be anonymised so I cannot be  identified. |  |
| 1. I understand that information collected will be managed by the study team only. The audio-recording and paper transcripts will be destroyed at the end of the study. The anonymised transcribed interview data will be destroyed after a period of fifteen years. |  |

**Name of staff participant Signature Date**

**Name of person taking consent Signature Date**
