# Supplementary material for: Nurses’ and patients’ experiences and preferences of the ankle-brachial pressure index and multi-site photoplethysmography for the diagnosis of peripheral arterial disease: A qualitative study
Source: PLoS One. 2019 Nov 7;14(11):e0224546. doi: 10.1371/journal.pone.0224546 (PMC6837749; doi:10.1371/journal.pone.0224546)
Supplement: S5 File — (DOCX) [file pone.0224546.s005.docx]

**Research Participant Consent Form – Patient Interviews**

**Novel pulse device for diagnosis of PAD (NOTEPAD)**

| **P** |  |  |  |
| --- | --- | --- | --- |

|  |  |
| --- | --- |

**Number:**

|  | **Please initial** |
| --- | --- |
| 1. I confirm that I have read and understand the information sheet   Version 2.0*,* dated 19 December 2014, for the above study. I have had the opportunity to ask questions and received satisfactory answers. |  |
| 1. I understand that my participation is voluntary and that I am free   to withdraw without giving any reason, without my medical care or legal  rights being affected. If I decide to withdraw, any data (information),  collected up to the point I withdraw, may be used in order to preserve  the value of the study. I understand that no further information will be  collected after my withdrawal unless I agree to an interview to discuss this. |  |
| 1. I agree to take part in a qualitative interview for the study. |  |
| 1. I understand and agree to the interview being audio recorded. |  |
| 1. I understand that I will not be personally named in any report and that   anything I say will be treated with confidence (unless I say something  that indicates that either myself or someone else is at risk of harm and  this would be discussed with me prior to telling anyone else) |  |
| 1. I understand that any information collected will be kept in a secure   way and that interview data will be anonymised so I cannot be identified. |  |
| 1. I understand that information collected will be managed by the study   team only. The audio-recording and paper transcripts will be destroyed at the end of the study. The anonymised transcribed interview data will be destroyed  after a period of fifteen years. |  |

**Name of Patient Signature Date**

**Name of person taking consent Signature Date**
