# Supplementary material for: Nurses’ and patients’ experiences and preferences of the ankle-brachial pressure index and multi-site photoplethysmography for the diagnosis of peripheral arterial disease: A qualitative study
Source: PLoS One. 2019 Nov 7;14(11):e0224546. doi: 10.1371/journal.pone.0224546 (PMC6837749; doi:10.1371/journal.pone.0224546)
Supplement: S6 File — (DOCX) [file pone.0224546.s006.docx]

**Interviews with case participants: Topic Guide**

**History of PAD**

What symptoms

Impact on life

**Diagnosis of PAD**

When diagnosed

How diagnosed

**Management of PAD**

How is PAD managed?

Prefer by GP or hospital?

Thoughts on management?

**Experience of being in study**

Understood reasons for research?

Was each test explained beforehand?

Lying down ok?

Amount of rest time ok?

**MPPG Device**

Time taken?

Comfort?

Confidence?

Concerns?

Did you expect to feel something when the device was recording? Would it have made a difference if you did?

**ABI Device**

Previous experience with it?

Time taken?

Comfort?

Confidence?

Concerns?

**Preference for tests?**
