# Supplementary material for: Nurses’ and patients’ experiences and preferences of the ankle-brachial pressure index and multi-site photoplethysmography for the diagnosis of peripheral arterial disease: A qualitative study
Source: PLoS One. 2019 Nov 7;14(11):e0224546. doi: 10.1371/journal.pone.0224546 (PMC6837749; doi:10.1371/journal.pone.0224546)
Supplement: S7 File — (DOCX) [file pone.0224546.s007.docx]

**Topic Guide**

**Interviews with Health Professionals**

|  |  |
| --- | --- |

- **BACKGROUND**
  - their role
- **TRAINING**
  - How were you trained
    - Timing In relation to use of device
    - Clarity
    - Sufficient for task (follow up interview)
    - Suggestions for improvement
    - ‘Real world’ what do people need to know?
    - ‘Real world’ what training do you get with other new devices?
- **VIEWS ON PPG**
  - Confidence in using device
  - Concerns about using device
  - Ease of use
  - Feedback from patients
  - Views on device out-put
  - Suggestions for improvement
- **VIEWS ON ABI**
  - Previous experience of PAD and ABI
  - Confidence in using device
  - Concerns about using device
  - Ease of use
  - Feedback from patients
  - Views on device out-put
  - Suggestions for improvement
  - Comparisons with PPG?
- **OTHER**
  - Why do you explain what you’re doing to patients? Eg put them at ease, or does it just reflect what you’re thinking (ie rehearsal)?
- **THOUGHTS ON FUTURE USE OF PPG**
