# Supplementary material for: Nurses’ and patients’ experiences and preferences of the ankle-brachial pressure index and multi-site photoplethysmography for the diagnosis of peripheral arterial disease: A qualitative study
Source: PLoS One. 2019 Nov 7;14(11):e0224546. doi: 10.1371/journal.pone.0224546 (PMC6837749; doi:10.1371/journal.pone.0224546)
Supplement: S8 File — (DOCX) [file pone.0224546.s008.docx]

| **No** | **Item** | **Guide questions/description** | **Included?** |
| --- | --- | --- | --- |
| **Domain 1: Research team and reflexivity** |  |  |  |
| Personal Characteristics |  |  |  |
| 1. | Interviewer/facilitator | Which author/s conducted the interview or focus group? | Yes; located in methods section |
| 2. | Credentials | What were the researcher's credentials? *E.g. PhD, MD* | Yes; as part of author information |
| 3. | Occupation | What was their occupation at the time of the study? | No; this journal does not display author occupations |
| 4. | Gender | Was the researcher male or female? | Yes; gender of researchers involved in data collection are stated in methods section |
| 5. | Experience and training | What experience or training did the researcher have? | Yes; demonstrated in author credentials |
| Relationship with participants |  |  |  |
| 6. | Relationship established | Was a relationship established prior to study commencement? | Yes; recruitment process for sites and participants is detailed in methods section |
| 7. | Participant knowledge of the interviewer | What did the participants know about the researcher? e*.g. personal goals, reasons for doing the research* | Yes; recruitment process for participants is explained in methods section, which included reasons for doing the research |
| 8. | Interviewer characteristics | What characteristics were reported about the interviewer/facilitator? e.g. *Bias, assumptions, reasons and interests in the research topic* | Yes; data analysis section explains how rigour was ensured, thus reducing potential for bias and/or assumptions |
| **Domain 2: study design** |  |  |  |
| Theoretical framework |  |  |  |
| 9. | Methodological orientation and Theory | What methodological orientation was stated to underpin the study? *e.g. grounded theory, discourse analysis, ethnography, phenomenology, content analysis* | Yes; methods section |
| Participant selection |  |  |  |
| 10. | Sampling | How were participants selected? *e.g. purposive, convenience, consecutive, snowball* | Yes; methods section |
| 11. | Method of approach | How were participants approached? e*.g. face-to-face, telephone, mail, email* | Yes; methods section |
| 12. | Sample size | How many participants were in the study? | Yes; methods section |
| 13. | Non-participation | How many people refused to participate or dropped out? Reasons? | Yes; methods section |
| Setting |  |  |  |
| 14. | Setting of data collection | Where was the data collected? e*.g. home, clinic, workplace* | Yes; methods section |
| 15. | Presence of non-participants | Was anyone else present besides the participants and researchers? | Yes; methods section |
| 16. | Description of sample | What are the important characteristics of the sample? *e.g. demographic data, date* | Yes; methods section |
| Data collection |  |  |  |
| 17. | Interview guide | Were questions, prompts, guides provided by the authors? Was it pilot tested? | Yes; methods section |
| 18. | Repeat interviews | Were repeat interviews carried out? If yes, how many? | No; participants were not offered repeat interviews. |
| 19. | Audio/visual recording | Did the research use audio or visual recording to collect the data? | Yes; methods section |
| 20. | Field notes | Were field notes made during and/or after the interview or focus group? | Yes; methods section |
| 21. | Duration | What was the duration of the interviews or focus group? | Yes; methods section |
| 22. | Data saturation | Was data saturation discussed? | Yes; methods section |
| 23. | Transcripts returned | Were transcripts returned to participants for comment and/or correction? | No; participants were not offered the opportunity to review transcripts. |
| **Domain 3: analysis and findings**z |  |  |  |
| Data analysis |  |  |  |
| 24. | Number of data coders | How many data coders coded the data? | Yes; methods section |
| 25. | Description of the coding tree | Did authors provide a description of the coding tree? | Yes; findings section |
| 26. | Derivation of themes | Were themes identified in advance or derived from the data? | Yes; methods section |
| 27. | Software | What software, if applicable, was used to manage the data? | Yes; methods section |
| 28. | Participant checking | Did participants provide feedback on the findings? | No; participants did not provide feedback on the findings. Nurse participants were invited to a seminar to discuss preliminary findings but as none chose to attend we did not report the invite in the manuscript. |
| Reporting |  |  |  |
| 29. | Quotations presented | Were participant quotations presented to illustrate the themes / findings? Was each quotation identified? e*.g. participant number* | Yes; findings section |
| 30. | Data and findings consistent | Was there consistency between the data presented and the findings? | Yes; findings section |
| 31. | Clarity of major themes | Were major themes clearly presented in the findings? | Yes; findings section |
| 32. | Clarity of minor themes | Is there a description of diverse cases or discussion of minor themes? | Yes; findings section |
